# Structural Formation of Soil Concretes Based on Loam and Fly Ash, Modified with a Stabilizing Polymer Additive

**DOI:** 10.3390/ma15144893

**Published:** 2022-07-14

**Authors:** Nataliya Konovalova, Pavel Pankov, Valery Petukhov, Roman Fediuk, Mugahed Amran, Nikolai Ivanovich Vatin

**Affiliations:** 1 Irkutsk State Transport University, 664074 Irkutsk, Russia; konovalovanatasha@rambler.ru (N.K.); pavelpankov110990@mail.ru (P.P.); 2 Far Eastern Federal University, 690922 Vladivostok, Russia; petukhov.vi@dvfu.ru; 3 Peter the Great St. Petersburg Polytechnic University, 195251 St. Petersburg, Russia; vatin@mail.ru; 4 Department of Civil Engineering, College of Engineering, Prince Sattam Bin Abdulaziz University, Alkharj 11942, Saudi Arabia; m.amran@psau.edu.sa; 5 Department of Civil Engineering, Faculty of Engineering and IT, Amran University, Amran 9677, Yemen

**Keywords:** utilization of industrial waste, fly ash, soil stabilization, road construction materials, soil concrete, stabilizing additive, structure formation

## Abstract

Finding new ways of recycling production waste to improve the characteristics of various building materials is an urgent scientific task. This article substantiates the possibility of the disposal of fly ash in the composition of soil concrete, which is used in the construction of the structural layers of road pavements, foundations of buildings and structures, as well as sites for various purposes. The scientific novelty lies in the fact that the structure formation of soil concretes based on loam and fly ash and modified with a stabilizing additive is being studied for the first time. It was found that the investigated fly ash, according to its hydraulic properties, is classified as latent active and can be introduced into the compositions of road soil concrete modified with additives of various resources. The effectiveness of the complex method of stabilization, due to changes in soil properties as a result of the use of the binding and stabilizing additives of polymer nature “Kriogelit”, is shown. It was found that the optimal content of binder and fly ash in the samples was 8 and 10 wt.%, respectively. It was established that the use of the stabilizing additive “Kriogelit” makes it possible to obtain soil concrete with the highest strength (compressive strength 2.5 MPa, flexural strength 0.5 MPa) and frost resistance of at least F15. The microstructure, the degree of dehydration and carbonization, and the phase composition of the initial raw mixtures and soil concretes stabilized with the addition of “Kriogelit” were studied by methods of scanning electron microscopy, X-ray diffraction analysis, differential scanning calorimetry, thermogravimetry, and infrared spectroscopy. It was shown that organo-mineral complexes, with the participation of polymer and montmorillonite, are formed in stabilized soil concrete. It was revealed that structure formation is accompanied by the physical adsorption of the polymer on active centers of silicate minerals, carbonization, and hydration–dehydration processes. It was found that the reason for the increase in the strength of stabilized soil concretes is the hydrophobization of the porous structure of minerals, as well as the formation of calcium oxide silicate and dicalcium hydrated silicate. By the method of performing biotests with the test objects *Daphnia magna *Straus and *Chlorella vulgaris* Beijer, it was proven that the developed road concretes modified with the stabilizing additive “Kriogelit” do not have an acute toxic effect on the test objects and are safe for the environment and human health.

## 1. Introduction

Currently, an important ecological problem is the global anthropogenic transformation of the natural environment caused by the intense impact of industry on natural ecosystems [1,2,3,4]. Reducing anthropogenic impact through the disposal of industrial waste is a priority area for the development of various industries [5,6,7]. The solution to this problem acquired particular importance for the enterprises of the fuel and energy complex, whose activities lead to significant changes in the earth’s surface, disruption of natural landscape complexes, the withdrawal of significant land areas, the development of erosion processes, as well as changes in the quality and regime of surface runoff [8,9,10,11,12]. A significant technogenic load is exerted by the dusting of ash and slag waste dumps, since highly dispersed fly ash can remain in the air for a long time and cause irreparable damage to human health, pollute soils, surface waters, snow cover, and also contribute to a change in the geochemistry of landscapes, soil degradation, and the formation of a technogenic soil-water horizon.

At the same time, ash and slag waste is a valuable secondary raw material for various industries; therefore, the development of methods for their disposal is an urgent task. Numerous studies indicate there is a demand for ash and slag waste in the construction industry for the production of bricks and ceramics [13,14,15,16], thermal insulation materials [17,18,19,20], independent binders or active additive to organic and inorganic binders [18,21,22], concrete [23,24,25,26], composite materials [27,28,29], etc.

Analysis of scientific and technical advances in the production of road building materials made it possible to identify ways of utilizing ash and slag waste in composites based on soils in combination with additives of various natures [30,31,32,33,34]. Good results in this direction were obtained by Correns [35], Herzod and Mitchell [36], Dunn and Salem [37], Hilt and Davidson [38], Laguros [39], and others. However, soils strengthened only with cement are characterized by high rigidity and cracking in the autumn-winter period. When liming soils, it is difficult to provide the required frost resistance. The use of organic binders promotes the development of plastic deformations in the base layer. To improve the properties of soils, the most effective methods can be complex strengthening methods that combine changes in soil properties as a result of the use of binders and various stabilizing additives.

Kianimehr et al. [40] researched recycled concrete aggregates to stabilize clay soils. Chen et al. [41] optimized an ash–slag geopolymer for use in stabilizing soft soils. Kulkarni and Mandal [42] evaluated the strength of soil stabilized with nanosilica-cement mixtures as a road building material. Huang et al. [43] conducted a comprehensive review of polymers for soil stabilization. Saldanha et al. [44] studied the technical and environmental performance of egg lime for ground strengthening. Gonzalez et al. [45] used wastewater treatment sludge, activated blast furnace slag, and biochar as a low carbon binder to stabilize soft soils. Zhang et al. [46] developed sustainable reclaimed binders using industrial solid waste to stabilize a ground. Renjith et al. [47] optimized soils with fly ash and secondary additives for sustainable road construction. Correa-Silva et al. [48] studied in detail the geomechanical behavior of soft soil stabilized by alkali-activated blast furnace slags. Zhang et al. [49] investigated the durability of recycled lignin-stabilized muddy soil for environmentally friendly engineering materials.

Safi and Singh [50] made an efficient and effective improvement on stabilized clay soil with waste materials. Zinchenko et al. [51] studied the efficient stabilization of soil, sand, and clay by a polymer network of biomass-derived chitosan and carboxymethyl cellulose. Abbey et al. [52] experimentally studied the use of RoadCem blended with by-product cementitious materials for the stabilization of clay soils. Pakharel and Siddiqua [53] researched the effect of calcium bentonite clay and fly ash on the stabilization of organic soil from Alberta, Canada.

The most effective way is the use of fly ash as an independent binder when strengthening sandy loam, gravel sand, and sandy soils [54,55]. In this case, structure formation, in comparison with soils treated with cement, proceeds slowly, and the strength characteristics of the samples are studied after 90–360 days of hardening. A qualitative difference is revealed in the processes of interaction with various binders and additives due to the large specific surface area of the dispersed parts of the waste [56,57]. When a stabilizer and a binder are introduced into the clay soil, physicochemical and colloidal processes take place, which make it possible to impart specified properties to road building composites [58,59,60]. It should also be noted that the problem of the involvement of inert fly ash in the composition of road soil concrete is clearly insufficiently studied. The scientific novelty lies in the fact that the structure formation of soil concretes based on loam and fly ash modified with a stabilizing additive is being studied for the first time. The superiority of this article lies in its comprehensive research, including the study of mechanical and durability characteristics with an explanation of their improvement through the study of scanning electron microscopy, X-ray diffraction analysis, differential scanning calorimetry, thermogravimetry, and infrared spectroscopy, as well as biotesting.

The aim of this work was to develop compositions of soil concrete based on loam and fly ash, modified with a stabilizing polymer additive “Kriogelit”, and to study the processes of their structure formation. To achieve this aim, the following tasks were completed: the study of the microstructure of samples using scanning electron microscopy, X-ray diffraction, derivation thermogravimetry, infrared spectra, and environmental safety research using biotesting.

The critical improvement from the application of research results lies in the development of environmentally friendly road construction materials based on large-scale production waste.

## 2. Materials and Methods

### 2.1. Characteristics of the Raw Materials Used

**Figure 1:**
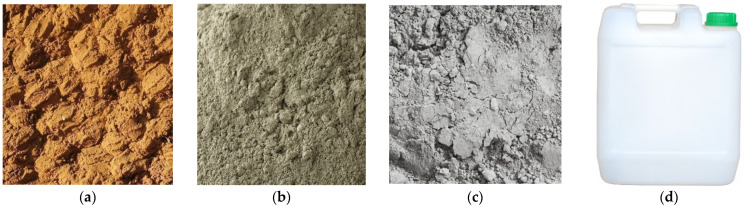
Appearance of the materials used: (**a**) loam, (**b**) fly ash, (**c**) Portland cement, and (**d**) stabilizing polymer additive.

Samples of soil concrete were obtained based on clay soil (loam) and fly ash (Chita, Russia). Portland cement with additives CEM II/A-S 32.5B (Angarsk, Russia) was used as a binder for the modification of mineral raw materials. To improve the performances of soil concrete, the stabilizing polymer additive “Kriogelit” was used, which is a transparent viscous liquid; the density, determined by the pycnometric method, is 1.20 g/cm^3^; and the reaction of the medium pH = 8. Figure 1 shows the appearance of the materials used.

**Figure 2:**
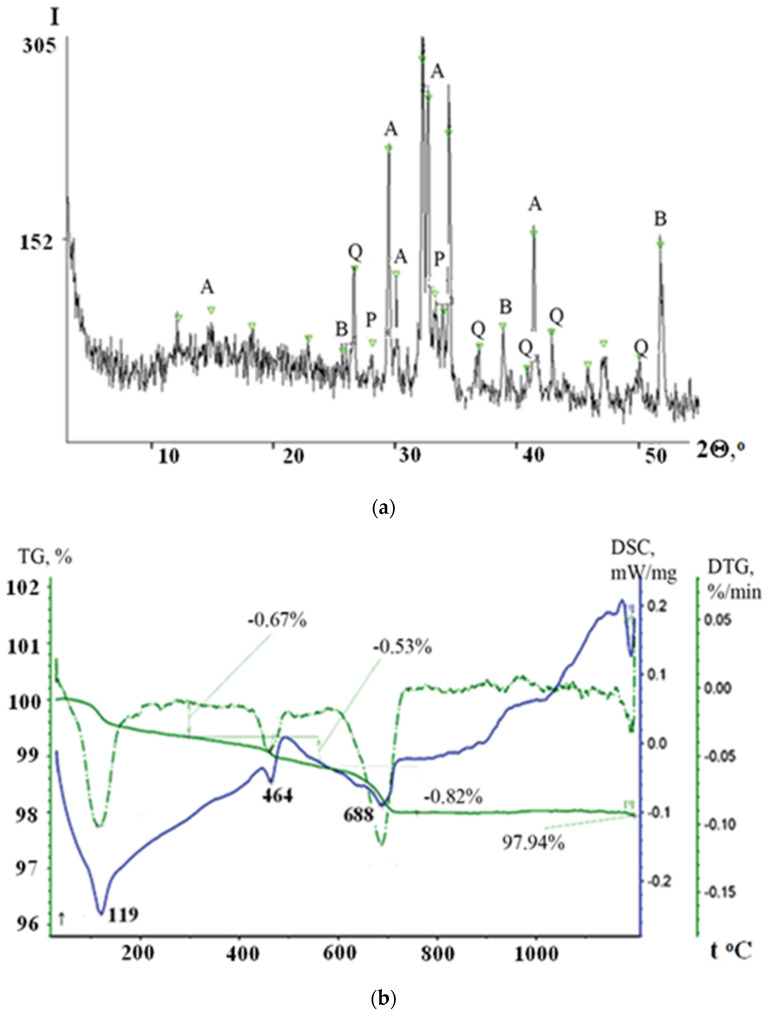
XRD (**a**) and TG (**b**) patterns of the Portland cement minerals and products: A—C_3_S; B—C_2_S; P—Ca(OH)_2_; Q—SiO_2_.

Figure 2 shows XRD and TG patterns of the Portland cement. Analysis of the data on the XRD pattern of the Portland cement (Figure 2a) reveals that it contains alite Ca_3_SiO_5_ (5.95; 3.03; 2.97; 2.74; and 2.18 Å), belite Ca_2_SiO_4_ (3.43; 2,88; 2.81; 2.28; and 1.76 Å), portlandite Ca(OH)_2_ (3.19; 2.65 Å), and quartz SiO_2_ (3.35; 2.44; 2.32; 2,21; 2.11; and 1.82 Å).

Analyses of DSC and TG patterns in the temperature range from 25 to 1200 °C allowed, establishing that calcite is the main phase of Portland cement (Figure 2b). The first stage of weight loss on the thermogravimetric curve, amounting to 0.67%, is due to the release of water. Dehydration of Ca(OH)_2_ occurs after 400 °C and reaches a maximum at 462 °C. At 1150 °C, CaSO_4_ melts and decomposes. The differential thermogravimetric curve exhibits an asymmetric extremum at 688.1 °C, indicating the presence of carbonate impurities. Thus, the weight loss during the dissociation of carbonates is 0.82%, while the calcite stage accounts for 0.59 and 0.23%.

The grain composition of fly ash is shown in Table 1. The properties of fly ash are presented in Table 2. According to the activity group, fly ash is latent active and does not exhibit binder properties. The specific surface of the particles is 276 m^2^/kg and the true density is 2240 kg/m^3^. The value of the specific activity of natural radionuclides (^226^Ra, ^232^Th, and ^40^K) is 248 Bq/kg, which makes it possible to use this waste in construction without restrictions.

**Figure 3:**
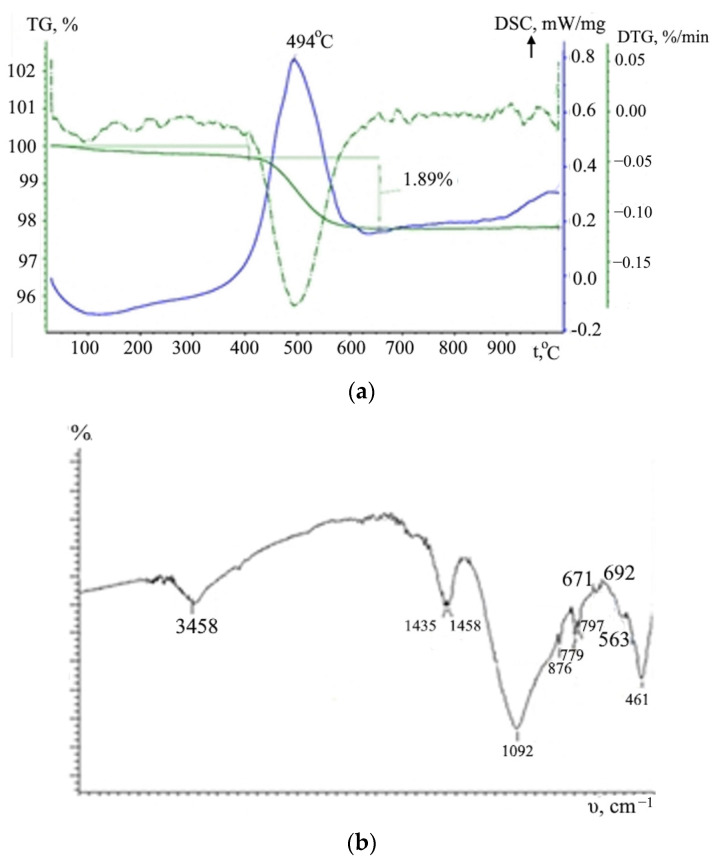
DSC and TG patterns (**a**) and IR (**b**) of the fly ash.

The mineralogical composition of fly ash is represented by quartz, calcite, feldspar, goethite, and the X-ray amorphous phase. Inspection of ash by DSC and TG methods reveal thermal activity of the sample (Figure 3a). The weight loss accompanying the exothermic effect with a maximum at 493.5 °C was 1.89%. There are no steps associated with the decomposition of carbonates; small jumps in the increase in the rate of weight loss are visible at 680 and 715 °C. When heated from 25 to 400 °C, the sample loses 0.3% of adsorbed moisture.

According to infrared spectroscopy data, the ash contains calcite, as evidenced by absorption bands with maxima at 1454; 1435 and 876 cm^−1^, which is associated with stretching and bending vibrations of the CO_3_^2−^ group (Figure 3b). The presence of quartz and crystobalite in the ash is evidenced by characteristic bands with maxima at 797; 779 cm^−1^ and 692; 671 cm^−1^, respectively. The bands at 3458 and 1092 cm^−1^ correspond to stretching vibrations of OH and Si-O-Si (Al) groups. Infrared spectrum of fly ash, ν cm^−1^: (νCO_3_^2−^) 1454, 1435; (δCO_3_^2-^) 876; (νSiO_2_) 797, 779; (νSiO_2_) 692, 671; (νOH) 3458; (νSi-O-Si(Al)) 1092; (δ Si-O-Si(Al)) 461; and (δFe-O-Fe) 563. The properties of the loam are shown in Table 3.

**Figure 4:**
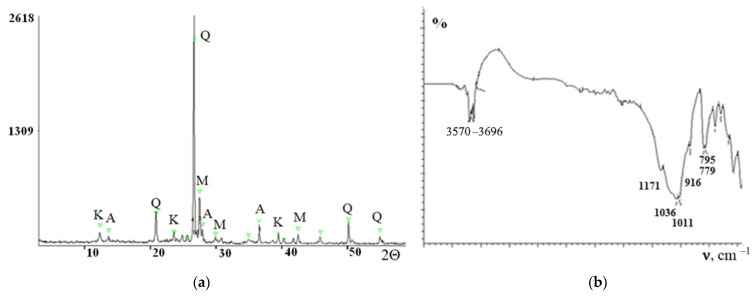
XRD pattern (**a**) and IR spectrum (**b**) of the loam. Q—quartz; A—albite; M—microcline; and K—kaolinite.

X-ray diffraction analysis of the loam (Figure 4a) made it possible to establish that its composition includes quartz SiO_2_ (4.26; 3.33; 1.82 Å); albite NaAlSi_3_O_8_ (6.48; 3.19; 2.45 Å); microcline KAlSi_3_O_8_ (3.29; 2.90; 2.16 Å); and kaolinite Al_2_ (Si_2_O_5_) (OH)_4_ (7.19; 3.70; 2.23 Å).

Numerous absorption bands with maxima in the range of 3570–3696 cm^−1^ in the IR spectrum of the loam (Figure 4b) indicate stretching vibrations of OH groups belonging to water. The absorption bands with maxima at 1011, 1036, and 1171 cm^−1^ belong to the Si-O-Si group. The band at 916 cm^−1^ corresponds to stretching vibrations of the Si-O-Al group, which corresponds to the mineral composition of loam. The doublet at 795 and 779 cm^−1^ is due to the presence of quartz in the composition of the loam. Infrared spectrum of soil, ν cm^−1^: (νSiO_2_) 795, 779; (νOH) 3570–3696; (νSi-O-Al) 916; and (νSi-O-Si) 1011, 1036, and 1171.

Table 4 shows the chemical composition of the raw materials.

In this way, the initial mineral raw material belongs to multiphase polymineral systems. In this regard, when modifying them with stabilizing additives that increase the operational characteristics of the final product, it is advisable to pay attention to the homogenization of finely crushed particles. It should be taken into account that the large specific surface area of dispersed parts of loam leads to a qualitative difference in the processes of their interaction with binders and stabilizing additives.

### 2.2. Mix Design

**Figure 5:**
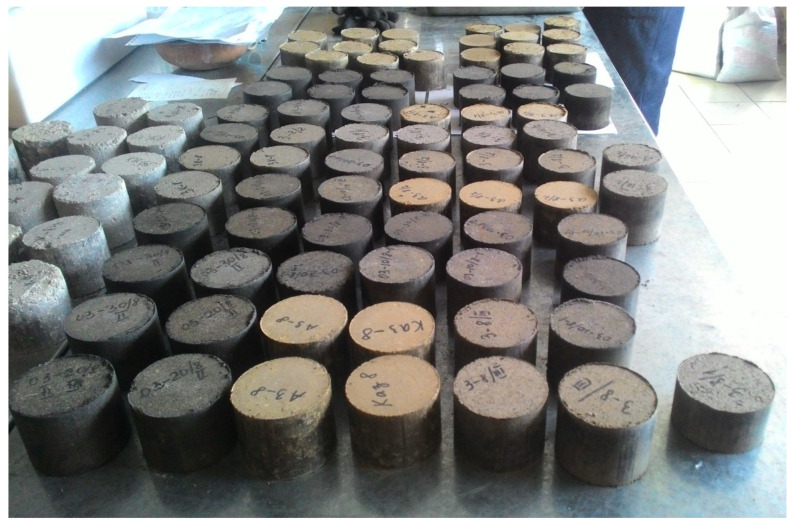
Soil concrete samples.

The dosages of fly ash and Portland cement in the soil concrete samples were determined experimentally. Samples in the form of cylinders with a diameter of 71.4 mm (Figure 5) and beams with dimensions of 160 × 40 × 40 mm were made and tested according to the standard procedure in accordance with the requirements of the Russian standard GOST 23558-94.

**Figure 6:**
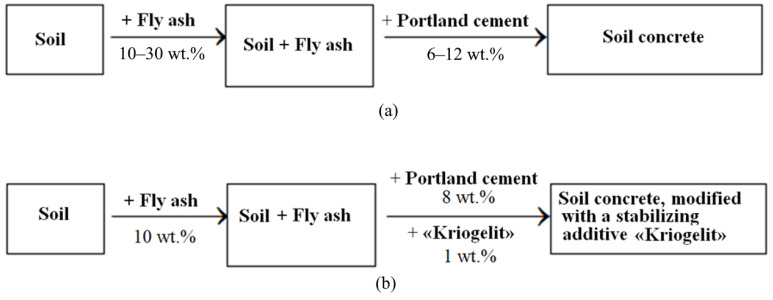
Technological routes for obtaining: (**a**) soil concrete based on loam, fly ash, and Portland cement; (**b**) soil concrete of optimal composition, modified with the stabilizing additive “Kriogelit”.

The mixture was compacted in steel molds with a compaction load of 15 MPa. Physicomechanical characteristics were studied on samples subjected to complete water saturation after 28 days. The technological route for obtaining soil concrete with additives of fly ash and Portland cement is shown in Figure 6a. To improve the performance of soil concrete with optimal dosages of raw materials, the samples were modified with a stabilizing additive of a polymer nature, i.e. “Kriogelit”. The technological scheme for producing soil concrete modified with the stabilizing additive “Kriogelit” is shown in Figure 6b.

Establishing the dependence of the physical and mechanical characteristics of the specimens on the content of Portland cement and fly ash will make it possible to regulate the strength of soil concrete. In order to establish the optimal compositions of soil concrete in the specimens, the weight fractions of Portland cement (6, 8, 10, and 12 wt.%) and fly ash (10, 20, and 30 wt.%) were varied (Table 5).

### 2.3. Laboratory Equipment and Research Methods

The production of specimen cylinders was carried out by a hydraulic press using hollow cylindrical molds with two inserts. The compaction load was selected so as to obtain the maximum density of the specimens, which is achieved at the optimum moisture content. The molds with the mixture were kept under load for 3 min. The height of the specimens was 70 ± 1.5 mm. Specimen beams were prepared by pressing in steel molds with double-sided inserts.

The compressive strength was determined on cylinder specimens of two groups: the first group of specimens was kept for 28 days in a bath with a hydraulic seal at full water saturation, the second was kept in air at a temperature of (20 ± 2) °C. Before testing the compressive strength, the specimens were immersed in water for 48 h; at first the samples were filled with water to 1/3 of the height for 6 h, and then water was added until they were completely immersed and kept for 42 h. A compressive strength was determined at a press platen speed of 3.0–0.3 mm/min.

The strength of soil concrete for three-point bending was determined on samples 150 × 150 × 600 mm in size. The load increased uniformly at a rate of 0.06 MPa per second.

To establish frost resistance, water-saturated specimens were loaded into a freezer and kept for 2.5 h at a temperature of minus (18 ± 2) °C. The specimens were placed at a distance of at least 50 mm from each other; the beginning of freezing was considered the moment when the temperature was set at minus 16 °C. The specimens were thawed in a water bath at a temperature of (18 ± 2) °C for 2 h, and the water layer above the specimens was at least 50 mm. The frost resistance grade was established by the number of freeze–thaw cycles, after which the strength of the specimens decreased by no more than 25%.

**Figure 7:**
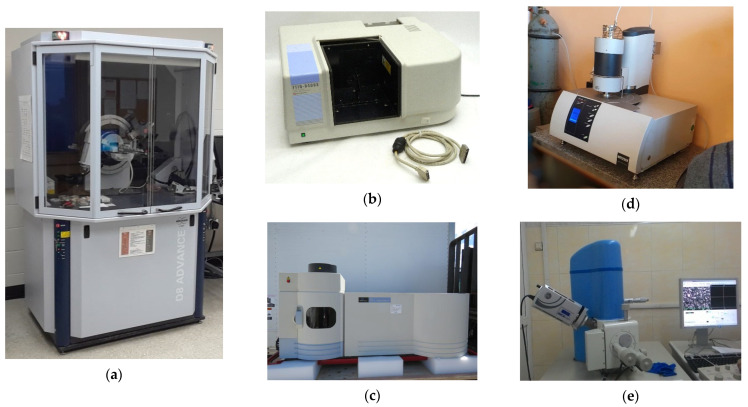
Laboratory equipment used: (**a**) D8 Advance X-ray diffractometer, (**b**) SHIMADZU FTIR-8400S infrared Fourier spectrometer, (**c**) Optima 5300DV emission spectrometer, (**d**) STA 449F1 synchronous thermal analyzer, and (**e**) JSM-6510LV JEOL scanning electron microscope.

X-ray diffraction analysis of the mineral raw materials was performed by powder diffraction using a D8 Advance X-ray diffractometer (Bruker AXS) (Figure 7a): goniometer radius—250 mm, radiation—CuK_α_, U = 40 kV, I = 40 mA, Goebel mirror, Soller slit—2.5 mm, scintillation counter, 2θ = 3–60°, scanning step—0.02°, and exposure—1 s/step. The mineralogical composition of the samples was deciphered using the EVA phase search program (Diffrac^plus^ PDF-2, 2007). The reference intensity ratio (RIR) method [61] was carried out for a semi-quantitative analysis of certain phases in the samples according to the corundum numbers of mineral phases from the PDF-2 crystallographic database (2007).

Infrared spectra were recorded with a FTIR-8400S infrared Fourier spectrometer (SHIMADZU, Tokyo, Japan) (Figure 7b) in the range of 4000–400 cm^−1^ on KBr tablets. The baseline method was used to calculate the relative intensity of absorption bands.

The chemical composition of mineral raw materials was determined by inductively coupled plasma atomic emission spectrometry performed by an Optima 5300DV emission spectrometer (167–403 nm) (PerkinElmer, Waltham, MA, USA) (Figure 7c). To determine the content of elements in the samples, the following schemes were used: ICP84T (fly ash); ICP84x, ICP61 (Portland cement); and ICP95A (loam).

Thermal analysis was performed using a STA 449F1 synchronous thermal analyzer (NETZSCH, Selb, Germany) (Figure 7d) by differential scanning calorimetry and thermogravimetry (DSC and TG). The specimens were heated from 30 to 998 °C in platinum crucibles in a dynamic argon atmosphere at a rate of 10 °C/min. The original thermogram files were recorded at a density of 100 points per minute and processed using the Proteus Analysis software (v 5.2.1) (NETZSCH, Selb, Germany).

The microstructure of the samples was examined with a JSM-6510LV scanning electron microscope (JEOL, Tokyo, Japan) with a microanalysis system, such as the INCA Energy 350 energy dispersive X-ray spectrometer (Oxford Instruments, High Wycombe, UK) (Figure 7e). A thin layer of platinum was deposited onto a non-conductive sample using a JFC-1600 setup.

The determination of the environmental safety of the materials developed during the study was carried out using the method of biotesting on the test objects *Daphnia magna *Straus and *Chlorella vulgaris* Beijer. The method for establishing toxicity is based on the change in the mortality and fertility of *Daphnia magna* Straus under the influence of toxic substances that may be present in the investigated aqueous extract from soil concrete, in comparison with a control sample that does not contain toxic substances. The acute toxic effect of the investigated aqueous extract was determined by the mortality (lethality) of the test object for a certain period of exposure. The criterion for acute toxicity was the death of 50% or more of the *Daphnia magna* Straus within 96 hours. In the control experiment, the death of test objects did not exceed 10%. Test conditions with the test object *Daphnia magna* Straus were as follows: pH = 7.0–8.5; t = 20 ± 2 °C; dissolved oxygen was, at the beginning of biotesting ≥ 6.0 mg/dm^3^, and at the end ≥ 2.0 mg/dm^3^; dilution ratio—0, 1, 10, and 100.

The assessment of the toxicity of the samples of aqueous extracts based on the change in the optical density of the test culture *Chlorella vulgaris* Beijer was carried out by the method of photoelectrocolorimetry. The criterion for the toxicity of the aquatic environment is a decrease of 20% or more (suppression of growth), or an increase of 30% or more (stimulation of growth) in the optical density of a culture of *Chlorella vulgaris* Beijer grown for 22 h in a tested aqueous extract, compared to its growth in a control medium prepared with distilled water. This makes it possible to establish the toxic concentration of individual substances or the toxic multiplicity of dilution of aqueous extracts at 22 h of light exposure in comparison with the control. Test conditions with the test object *Chlorella vulgaris* Beijer were as follows: pH = 7.0–8.5; t = 36.0 ± 0.5 °C.

## 3. Results and Discussion

### 3.1. Physical and Mechanical Properties

The dependence of the physical and mechanical properties of soil concrete specimens, according to Table 5, on the weight fraction of mineral raw materials (fly ash and Portland cement) is shown in Table 6. Analysis of the strength characteristics of the specimens showed a decrease in the compressive strength and flexural strength with an increase in the proportion of fly ash in the system. The optimum proportion of fly ash was 10%. Compressive strength at 20 °C is a maximum value for specimens containing 8 wt.% Portland cement. With a weight fraction of Portland cement of 12%, most of the soil concrete with 30 wt.% fly ash was destroyed at full water saturation. A decrease in the frost resistance of the specimens of any composition with an increase in the mass fraction of Portland cement in them was noted.

This was due to the fact that the clay, ash, and cement components of the samples have significant water absorption. An increase in the number of freeze–thaw cycles led to a loosening and decrease in the strength characteristics of the specimens. It was found that in composites containing optimal dosages of ash (ω = 10 wt.%) and Portland cement (ω = 8 wt.%), the density and specific surface area were ρ = 2.36 g/cm^3^, S_sp_ = 1230 cm^2^/g.

In order to make improvements in the strength and frost resistance of the soil concrete with an optimal composition (ω_ash_ = 10 wt.%), the efficiency of using the stabilizing additive “Kriogelit” (ω = 1.0 wt.%) was studied. The maximum strength characteristics of the specimens were achieved with a mass fraction of Portland cement of 8 wt.% (Table 7, specimen 5). The introduction of a stabilizing additive into the composition of specimens 1, 5, 9, and 13 promoted an increase in the strength and frost resistance of the composites.

The optimal dosage of Portland cement and fly ash in soil concrete compositions was established experimentally by the values of compressive and flexural strength under capillary and full water saturation at 20 °C, as well as frost resistance grades. Analysis of the strength characteristics of the samples showed that the optimal dosage of fly ash and Portland cement is 10 and 8 wt.%, respectively. There is a decrease in compressive and flexural strength with an increase in the proportion of fly ash in the system up to 30 wt.% both at capillary and at full water saturation.

### 3.2. Scanning Electron Microscopy

Soil concretes are heterophase multicomponent systems, the microstructure of which determines the strength characteristics. A stabilizing additive of a polymeric nature can interact with clay minerals and promote the formation of secondary soil microconglamerates. In this case, the presence of smectites with sliding elementary layers allow for polymer macromolecules to displace water molecules and contribute to the compaction of the polymer–inorganic composite.

**Figure 8:**
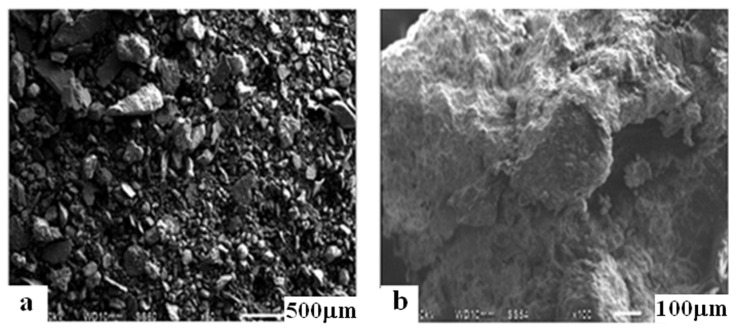
SEM images; (**a**) raw mix; and (**b**) soil concrete stabilized with additive «Kriogelit» (ID 5).

A special role in the processes of the formation of the structure of soil concrete in the presence of Portland cement and the stabilizing additive is assigned to the peculiarities of the morphology of clay particles. Microscopic studies of the raw mixture for manufacture of the specimen 5, as well as soil concrete of the same composition, stabilized with the addition of “Kriogelit” after 28 days of hardening, are shown in Figure 8.

Scanning electron microscopy images make it possible to establish that the initial raw material mixture (Figure 8a) is represented by rounded and acute-angled grains, as well as fragments of quartz, mica, feldspars, and other minerals. The raw mixture consists of polydisperse fractions of large particles of irregular shape with a size of 77–197 µm. Stabilization of soil concrete with Kriogelit promotes the formation of large agglomerates, ranging in size from 1.619 to 2.603 mm (Figure 8b). As a result of structure formation, the microstructure of the specimens takes the form of a single dense mass of large agglomerates, which is consistent with the data on the formation of a crystallization-coagulation structure in the compositions of soil concretes.

### 3.3. X-ray Diffraction

**Figure 9:**
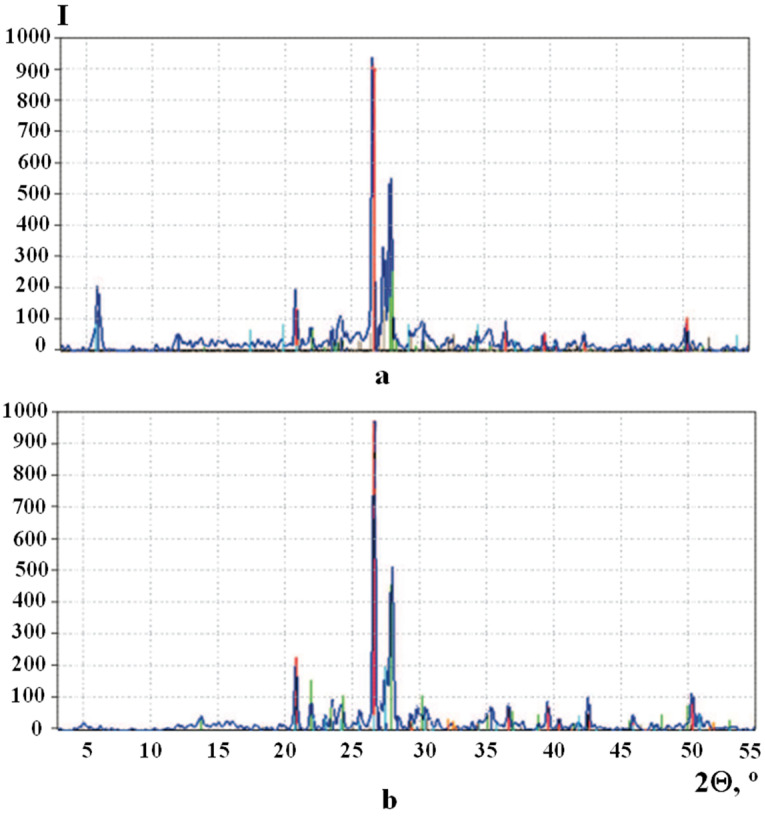
XRD patterns of soil concrete stabilized with additive «Kriogelit» (ID 5): (**a**) raw mix; (**b**) 28-day specimen.

The formation of new phases in the structure of soil concrete stabilized by polymer additives should be accompanied by the appearance of reflections that are absent in the diffraction patterns of the initial raw mixtures. In this regard, the samples were studied by powder diffraction. Diffraction patterns of the original and stabilized samples are shown in Figure 9.

The analysis of diffraction patterns made it possible to establish the mineralogical composition of the raw mixture and soil concrete stabilized with the addition of “Kriogelit”, ω, %:

- Raw mix (Figure 9a): 36.84 albite; 34.46 microcline; 8.15 calcium oxide silicate; 13.30 montmorillonite; and 7.26 quartz;
- Stabilized soil concrete (Figure 9b): 43.21 albite; 30.26 quartz; 19.08 microcline; 3.76 calcium silicate; and 3.69 dicalcium hydrated silicate.

The disappearance of montmorillonite reflections (d = 1.485; 0.740 nm) in the diffractogram of stabilized soil concrete (Figure 9b) indicates the intercalation of the polymer into the structure of this mineral, which contributes to an increase in the adhesion strength of the composites. It was found that, as a result of the participation of a representative of feldspar microcline in the structure formation, its content decreases by 1.8 times.

The results obtained indicate that the reason for the increase in the strength of the samples is the formation of calcium oxide silicate Ca_3_(SiO_4_)O, calcium silicate Ca_3_SiO_5_, and dicalcium hydrated silicate Ca_2_SiO_4_·H_2_O upon the interaction of silicates with Ca(OH)_2_.

### 3.4. Derivation Thermogravimetry

**Figure 10:**
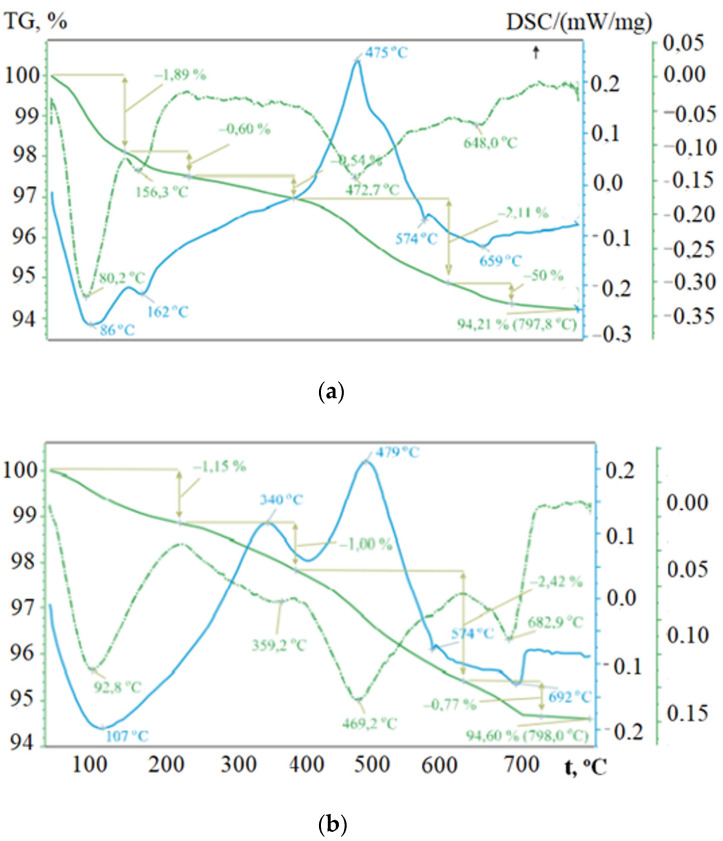
DTG patterns: (**a**) raw mix; (**b**) soil concrete stabilized with additive «Kriogelit» (ID 5).

An increase in the strength of soil concrete can occur as a result of carbonization. By treating them with carbon dioxide, as well as hydrating cement, therefore, it is convenient to study these processes by DSC and TG methods. Analysis of the DSC curves of the initial raw material mixture and stabilized soil concrete on its basis showed the presence of several thermal effects (Figure 10).

The endothermic effect with an extremum at 86 °C is caused by the removal of hygroscopic water, and at 162 °C by dehydration of the interlayer (adsorption) water of montmorillonite (Figure 10a). The endothermic effect at 574 °C is due to the polymorphic transformation of β-quartz to α-quartz. The only exothermic effect, which is recorded at 475 °C, arises from the burnout of a finely dispersed carbonaceous substance in the fly ash. On the thermogram of the fly ash, this exothermic effect is observed at 494 °C. The thermal effects listed above are characterized by losses on the TG mass curve, as well as four distinct extrema on the DTG curve.

Temperature shifts of endothermic effects at 107 and 692 °C on the DSC curve of the stabilized soil concrete (Figure 10b), explained by the processes of dehydration and decarbonization, indicate the hydrophobic activity of the Kriogelit additive due to the physical adsorption of polymer macromolecules on active centers of silicates. The endothermic effect observed on the thermogram of the raw mixture at 162 °C (Figure 10a) is absent in the specimen stabilized with the addition of “Kriogelit” (Figure 10b). The shape of the endothermic effect with an extremum at 107 °C is wide and has a blurred appearance.

The TG curves are also different, despite the fact that the total weight loss at t = 798 °C for the raw mixture and the stabilized soil concrete based on it have close values of 5.79 and 5.40%, respectively. It was found that, in comparison with the initial raw mixture, at first the weight loss occurs more slowly (up to a temperature of 360 °C), and then faster. The appearance on the DSC curve of the exothermic effect at 340 °C with a loss of mass on the TG curve is due to the decomposition of organic compounds bound into organomineral complexes (Figure 10b). In addition, the thermogram shows a shift and an increase in the intensity of the endothermic effect, at 659 °C (Figure 10b), caused by the decomposition of magnesium carbonate, up to 692 °C (Figure 10b). This is due to the additional carbonization of organic mineral soil concrete, which contributes to hardening and prevents the leaching of calcium hydroxide, as well as the erosion of cement stone. The absence of reflections belonging to magnesite in the diffractograms may indicate its X-ray amorphous structure.

The formation of colloidal “calcium-polymer compounds” in soil concretes is characterized by poor water retention. In this case, the thermogram and diffractogram should show thermal effects and reflections related to new growths and absent in the initial materials, which is not observed in this case (Figure 9 and Figure 10). Thus, using the DSC and TG methods, it was established that in the process of the structure formation of soil concretes, carbonization takes place, contributing to their strengthening. Clay minerals interact with the additive of the polymer nature “Kriogelit”, forming organo-mineral complexes.

### 3.5. Infrared Spectroscopy

**Figure 11:**
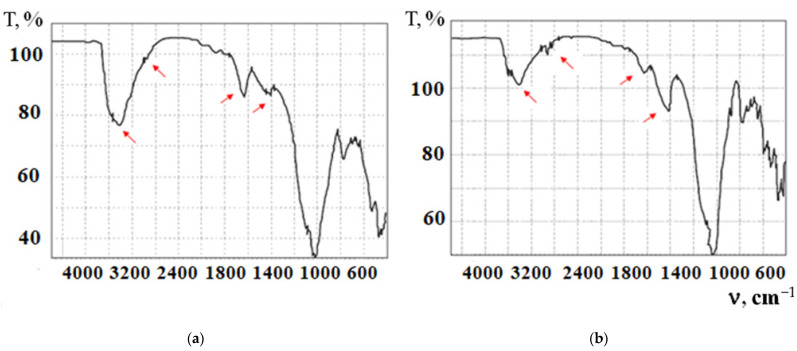
Infrared spectra of soil concrete stabilized with additive «Kriogelit» (ID 5): (**a**) raw mix; (**b**) 28-day specimen.

The structure of the raw mix and the stabilized soil concrete obtained on its basis was studied by infrared spectroscopy (Figure 11).

In the infrared spectrum of the initial raw material mixture (Figure 8a), absorption bands of crystallization water are observed in the range of ν = 3600–3400 and δ = 1634 cm^−1^, the intensities of which decrease after the addition of the stabilizing additive “Kriogelit”. This is due to the displacement of water molecules by the polymer and the blocking of active hydrophilic centers of smectites. New absorption bands are recorded at 2926 and 2855 cm^−1^, characteristic of the CH groups of the polymeric organic additive. The hydrophobizing activity of the additive “Kriogelit” is visible due to the decrease in the intensity of the absorption bands of stretching and bending vibrations of OH groups in interlayer water, which is associated with the penetration of stabilizer macromolecules into the montmorillonite gallery.

It was found that the intensity of absorption bands in the region of 1410–1420 cm^−1^ stretching vibrations of the CO_3_^2−^ group are enhanced in the spectrum of stabilized soil concrete, and an absorption band of deformation vibrations of this group appears at 878 cm^−1^, which indicates the ongoing carbonization process. The absorption band at 3622 cm^−1^ belongs to the Si-OH groups and indicates the hydroxylation of the mineral surface. A shift of the absorption bands of Si-O-Si is observed; OH and CO_3_^2−^ groups with a change in their intensity in the spectrum of stabilized soil concrete (Figure 11b) indicate the processes of physical adsorption of the polymer on active centers of silicates.

The emergence of a three-dimensional framework of dispersed particles interconnected through thin layers of water and polymer is facilitated by the introduction of the stabilizing additive “Kriogelit”.

### 3.6. Biotesting

The study of the environmental safety of soil concrete modified with the stabilizing additive “Kriogelit” was carried out using the biotesting method on the test objects *Daphnia magna* Straus and *Chlorella vulgaris* Beijer. The results of the biotesting of soil concrete modified with the stabilizing additive “Kriogelit” are shown in Table 8.

The biotesting data convincingly testify to the safety for the environment and human health of road soil concrete based on loam and fly ash, modified with the stabilizing additive “Kriogelit”. The developed soil concretes can be used in the construction of structural layers of road pavements, foundations, foundations of buildings and structures, as well as sites for various purposes.

## 4. Conclusions

This study is aimed at developing compositions of soil concrete based on loam and fly ash, modified with a stabilizing polymer additive “Kriogelit”, and studying the processes of their structure formation. Based on the results, the following conclusions were made:

- The fundamental possibility of using inactive fly ash for the composition of soil concrete was established as an effective way to solve a set of environmental problems.
- The dependence of the strength characteristics of soil concrete based on loam on the mass fraction of binder and fly ash was shown. It was found that to obtain composites with maximum strength, the optimal content of fly ash and Portland cement was 10 and 8 wt.%, respectively. It was established that the use of the stabilizing additive “Kriogelit” makes it possible to obtain soil concrete of the highest strength (compressive strength 2.5 MPa, flexural strength 0.5 MPa), and frost resistance of at least F15.
- It was revealed that the stabilizing additive “Kriogelit” promotes the formation of secondary soil microconglamerates and dense coagulation structure in soil concrete. In the process of structure formation, a crystallization framework of calcium silicate hydrates and calcium oxide-silicates is formed, as well as a coagulation framework of clay and dusty particles interconnected through thin layers of water and polymer. Carbonization and the formation of organomineral complexes during polymer intercalation to montmorillonite contribute to structure formation. According to its effect on the soil, the stabilizing additive “Kriogelit” is a “stabilizer-hydrophobizator”, which manifests itself in the blocking of active hydrophilic centers of smectites and quartz. In order to increase the adsorption capacity of the stabilizing additive, the minerals of the original raw mixture can be arranged in the following order: montmorillonite > microcline (feldspars) > quartz > albite.
- The ecological safety of soil concrete modified with the stabilizing additive “Kriogelit” was established. It was proven by the biotesting method that the developed soil concretes do not have an acute toxic effect on test objects (*Daphnia magna* Straus; *Chlorella vulgaris* Beijer) and are safe for the environment and human health.

Potentially, the application of the research lies in the development of environmentally friendly road building materials based on large-scale production wastes. Prospects for further research can be aimed at studying the features of the restoration of damaged and degraded ecosystems after the elimination of the waste dumps of enterprises.

## Figures and Tables

**Table 1:** Particle size distribution of the fly ash.

Remains on Sieves, % by Weight	Diameter of Holes of Control Sieves, mm						
	1.25	0.63	0.315	0.14	0.08	0.071	<0.071
partially	0.32	2.42	41.65	19.17	6.22	3.52	26.72
full	0.32	2.74	44.39	63.55	69.77	73.28	100.00

**Table 2:** Properties of the fly ash.

Properties	Values
Humidity, %	0.59
Bulk density, kg/m^3^	660
True density, kg/m^3^	2240
Specific surface area, m^2^/kg	276
Total remainder of the ash fraction, %	25.39
Bituminous capacity, g	54
Basicity module *M*_0_	0.17
Silicate module *M_c_*	1.82
Quality factor *K*	0.58
CaO_total_*,* wt.%	9.24
CaO_free_, wt.%	0.40
Activity group	hidden-active

**Table 3:** Properties of the loam.

Properties	Designations, Units of Measurement	Values
Soil density	ρ, g/cm³	2.0
Density of soil particles	ρ_s_, g/cm³	2.7
Density of dry soil	ρ_d_, g/cm³	1.6
Clay mineral content	ω, wt.%	39.0
Porosity coefficient	e, -	0.7
Natural soil moisture	W, fractions of a unit.	0.3
Moisture at the pour point	W_L_, fractions of a unit.	0.4
Moisture at the rolling edge	W_p_, fractions of a unit.	0.2
Plasticity number	I_p_, fractions of a unit.	0.2
Indicator of fluidity	I_L_, fractions of a unit	0.1
Specific adhesion	c, kPa	29
Angle of internal friction of soil	*φ*, °	26
Soil deformation modulus	E, MPa	25

**Table 4:** Chemical composition of the raw materials, %.

Raw Material	Al_2_O_3_	CaO	Fe_2_O_3_	MgO	SiO_2_	SO_3_	TiO_2_	Other	LOI
Portland cement	7.4	40.8	4.2	3.9	27.9	2.8	-	13.0	-
Fly ash	21.0	9.4	9.0	1.3	53.0	1.0	1.0	2.5	1.8
Loam	13.0	0.3	0.9	0.3	66.0	-	0.2	14.3	5.0

**Table 5:** Mix proportions, %.

ω, wt.%	Mix ID															
	1	2	3	4	5	6	7	8	9	10	11	12	13	14	15	16
Soil	84	74	64	94	82	72	62	92	80	70	60	90	78	68	58	88
Portland cement	6	6	6	6	8	8	8	8	10	10	10	10	12	12	12	12
Fly ash	10	20	30	0	10	20	30	0	10	20	30	0	10	20	30	0

**Table 6:** Mechanical and durability properties.

Properties	Mix ID															
	1	2	3	4	5	6	7	8	9	10	11	12	13	14	15	16
Compressive strength (hardening in water), MPa	1.6	1.5	1.4	1.6	1.7	1.7	1.7	1.7	1.7	1.6	1.3	1.7	0.8	0.7	0.6	1.0
Compressive strength, (hardening in air), MPa	1.7	1.6	1.6	1.8	2.2	2.2	2.0	2.5	2.0	2.1	1.8	2.5	1.8	1.7	1.5	1.9
Flexural strength, MPa	0.4	0.5	0.4	0.5	0.5	0.5	0.4	0.5	03	0.3	0.2	0.3	0.2	-	-	0.3
Frost resistance grade	F5	F0	F5	F5	F10	F5	F5	F5	F5	F5	F0	F10	F5	F5	F0	F10

**Table 7:** Mechanical and durability properties of the soil concrete stabilized with the additive “Kriogelit” (ω = 1.0 wt.%) at ω_ash_ = 10 wt.%.

Mix ID	Compressive Strength, MPa		Flexural Strength, MPa		Compressive Strength, (at Air Hardening), MPa	Frost Resistance Grade
	Water Saturation					
	Capillary	Full	Capillary	Full		
ID 1 with 1% Kriogelit”	2.02	2.08	1.08	1.04	3.69	F15
ID 5 with 1% Kriogelit”	2.91	2.54	1.28	1.36	3.92	F15
ID 9 with 1% Kriogelit”	2.50	2.40	1.42	1.42	3.12	F15
ID 13 with 1% Kriogelit”	2.65	2.38	1.50	1.48	3.00	F15

**Table 8:** Results of biotesting of soil concrete modified with the stabilizing additive “Kriogelit” (volume of water extract 1 dm^3^, dry residue of water extract 240 ± 22 mg/dm^3^).

Indicators		Test Objects	
		*Daphnia magna* Straus	*Chlorella vulgaris* Beijer
Duration, Hour		96	22
Biotesting results		Mortality to control, %	The relative difference in the average value of optical density for each dilution in comparison with the control, %
Dilution ratio	1	6.5	4.2
	10	3.1	7.4
	100	3.0	14.0
Sample evaluation		does not have an acute toxic effect	
